# Supplementary material for: Global shifts in mammalian population trends reveal key predictors of virus spillover risk
Source: Proc Biol Sci. 2020 Apr 8;287(1924):20192736. doi: 10.1098/rspb.2019.2736 (PMC7209068; doi:10.1098/rspb.2019.2736)
Supplement: Supplementary Information (SI) [file rspb20192736supp1.docx]

**Electronic supplementary materials for Global shifts in mammalian population trends reveal key predictors of virus spillover risk**

Christine K. Johnson, Peta L. Hitchens, Pranav S. Pandit, Julie Rushmore, Tierra Smiley Evans, Cristin C.W. Young, and Megan M. Doyle

**Expanded methods**

*Zoonotic Virus and Host Datasets*

Data were collected on zoonotic viruses and their terrestrial mammalian hosts published in English in the peer-review literature through December 2013. An initial list of viruses was established using Web of Science searches for topic keywords (zoonotic, zoonoses, and infectious animal disease, emerging wildlife disease), which were then cross checked with previously published summaries of human infectious diseases (1, 2). Virus-specific searches using the virus scientific name(s) and common name(s) were then conducted using the Web of Science database and reports were reviewed to gather data on mammal species implicated as a host of zoonotic viruses and criteria for implicating a species as a host in studies published through December 2013. Human viruses that have been recognized in animals (reverse zoonoses) were excluded.

Mammalian hosts investigated included all extant wild terrestrial mammalian species recognized by The International Union for Conservation of Nature (IUCN) Red List of Threatened Species (3). Marine mammal species were excluded as potential hosts to restrict analyses and inferences to terrestrial mammals. Domesticated species were included and categorized as an additional group to evaluate the impact of domestication on the number of viruses shared with humans. Mammals classified as domesticated species were sheep, goats, cattle, pigs, horses, Arabian camels, llamas, alpacas, donkeys, water buffalo, dogs, and cats. Given global population sizes and the vast literature investigating infectious diseases of domestic species, all domesticated species were classified as least concern, increasing and not data deficient.

Virus detections in mammalian species published in peer-reviewed literature were used to classify each species as a putative host or non-host for each virus. Both molecular techniques (PCR, virus isolation) and serologic techniques were included as evidence for a species to serve as a virus host. Molecular techniques tend to be more specific in identification of viruses in host species, while serological techniques tend to be less specific, but more sensitive in that they can detect previous exposure over an individual’s lifetime. Serologic techniques detect previous exposures to viruses and do not rely on identification of a very short period of viremia or virus shedding for detection, but rely on antibodies that can be cross-reactive, especially for genetically related viruses. Typically, peer-reviewed publication of virus findings by serology involves review of the evidence that the serologic test has been optimized to detect the specified virus or a cross-reacting genetically-related near neighbor. Therefore under-reporting of virus-host associations, or false negative virus-host associations, remains a predominant concern, given challenges in detection and the limited surveillance activities conducted to date relative to the number of virus-host associations possible across all terrestrial mammalian species. Because the purpose of this study was to estimate species-specific zoonotic virus richness, based on the sum of the number of zoonotic viruses detected in each species, we were equally concerned with both false negative and false positive host-virus associations, and thus included all reliable virus-host associations reported in the literature, regardless of the method used to detect the virus. Notably, the inclusion or exclusion of host serology data for model training in a recent study using machine learning to predict flavivirus wildlife reservoirs did not affect results regarding which wildlife species were likely to host select flaviviruses (4). Similarly, Olival et al. (5) found that including or excluding serological data when quantifying virus richness per mammalian species did not alter predictions of how host and virus traits affect patterns of virus spillover from wild mammals to humans, supporting the premise that misclassification of host status related to detection method is likely to be non-differential. The dataset of animal species associated with zoonotic viruses in this study is provided in Data file S1, which should be considered baseline data pending further investigations to confirm wild animal species as competent reservoirs or determine that some suspected host species are not epidemiologically important sources of spillover to humans. We propose re-evaluating epidemiological models of spillover risk in light of new data.

The IUCN Red List Criteria version 3.1 (3) used standardized criteria measured for species evaluated for Threatened status. These criteria were further evaluated for their relationship to zoonotic virus richness as a reflection of the probability of animal-human contact, given the species status. Criteria specifically evaluated in multivariable regression modeling included: 1) population size reductions (criteria A1- 4 by sub-criteria a, b, c, d, e); 2) limited geographic range (criteria B1 and B2); 3) small population sizes and declines (criteria C1 and C2); and 4) very small or restricted populations (D1 and D2) (3). Threatened categories were collapsed (i.e., Vulnerable, Endangered, and Critically Endangered were combined) to create new variables representing species ‘exposure’ to each criterion used for listing. Species listed with criterion A1-A4 were further collapsed across these categories and variables were evaluated for their ‘exposure’ to sub-criteria (a) direct observation (A1, A2, A4 only), (b) index of abundance appropriate to the taxon, (c) decline in area of occupancy, extent of occurrence, and/or quality of habitat, (d) actual or potential levels of exploitation, and (e) the effects of introduced taxa, hybridization, pathogens, pollutants, competitors or parasites, as described by the IUCN Red List Criteria version 3.1 (3). Threatened species listed in the IUCN Red List categories B1, B2, C1, C2, D1, and D2 were evaluated for exposure to these main criteria. All categories of criteria shown in Fig 2, regardless of hypothesized potential to reflect animal-human interactions, were evaluated for their relationship with zoonotic virus richness in a given species in multivariable modeling.

*Multivariable model selection*

Multivariable zero-inflated Poisson (ZIP) regression modeling was used to factors related to zoonotic virus richness (sum of zoonotic viruses) in each mammalian species. Both terms for reporting bias, log of the number of PubMed publications and data deficiency/unknown population trend, were evaluated in the ZIP model as an inflate variable (to predict excess zeros) and as a main effect variable, and selected based on optimization of model fit. Taxonomic order (n = 28 orders) was evaluated as a clustered random effect with robust standard errors and as a main effect to account for phylogenetic correlation among species within an order. The base mode included log number of PubMed publications, and variables were entered into the model by forward stepwise entry with all categories of a variable being entered at one time, starting with species status categories, then criteria for listing, then domestication status. Backward stepwise elimination was used to remove categories of variables with P <0.3. Two-way interactions between main effects that were significant in the multivariable model were similarly retained if significant (P ≤ 0.3). Taxonomic orders were entered in a forward stepwise manner in the model without order as random effect. Variables were retained in the final model if statistically significant (P ≤ 0.05), if they modified the coefficients of other covariates by more than 10% (indicating confounding), or if they improved overall model fit based on ∆deviance. Model sensitivity to order of variable inclusion through forward selection and backward elimination was evaluated by comparing terms retained in models using both approaches.

We assessed change in AIC and BIC scores to compare nested models and arrive at a best-fit minimal model that included significant independent variables and best explained variation in the sum of zoonotic viruses in a species. Overall model fit for the final model and the alternate best model was reported as McFadden’s R^2^. Competing multivariable models were evaluated by McFadden’s R^2^ and the Vuong test to compare overall model fit for non-nested models (Poisson, negative binomial, zero-inflated negative binomial, and other hurdle models). The best fit alternate model was a zero-inflated negative binomial model with log pubmed hits as the inflate variable, data deficient variable as a main effect. This alternate zero-inflated negative binomial model included all variables as significant effects, except the term for ‘population size reduction by direct observation’ was only marginally significant. The best fit alternate zero-inflated negative binomial model is shown in Table S2. We also show the best fit zero-inflated Poisson model without the variable representing number of publications in PubMed to indicate the influence reporting bias had on other variables related to virus richness in a species (Table S3).

**Linear correlation between species conservation status and zoonotic virus richness**

We evaluated the linear relationship between conservation status and zoonotic virus richness by converting ordered categories of conservation status into a numerical value and assessing the overall correlation between conservation status and zoonotic virus richness in a species using the non-parametric Spearman’s rho statistic, for species not described as data deficient (n = 3117). The numerical scale of conservation status had a positive linear relationship with the number of zoonotic viruses reported in a species. Specifically, species with increasing population trends had more zoonotic viruses (without data deficient species; rho = 0.215, two-sided P < 0.001).

**Supplementary tables**

**Table S1.** Ranking of variable groups from the zero-inflated Poisson regression model predicting the number of zoonotic viruses in a species. The change in AIC (∆AIC) values for the zero-inflated Poisson regression model is shown for removal of each variable group from the best-fitting (full) model: Zoonotic Virus Richness ~ Number of PubMed Publications(log) + Domesticated Species + Conservation Status [Least Concern increasing + Least Concern decreasing + Near Threatened decreasing + Vulnerable + Endangered + Critically Endangered] + IUCN Criteria for Threatened Status [Population size reduction by direct observation (A1,A2,A4(a)) + Decline in area of occupancy or habitat quality (A1-4(c)) + Population size reduction based on levels of exploitation (A1-4(d)) + Small extent of occurrence (B1)] + Taxonomic Order [Chiroptera + Diprotodontia + Eulipotyphla + Primates] + inflate (Data Deficient).

| **Variable groups** | **AIC** | $\Delta AIC$  **(**${AIC}_{fitted}-{AIC}_{full}$**)** |
| --- | --- | --- |
| Full model | 4416.753 | - |
| -log of PubMed publications for each species | 5592.514 | 1175.761 |
| -Conservation Status | 4572.182 | 155.429 |
| -Taxonomic order | 4562.084 | 145.331 |
| -IUCN criteria for threatened species | 4523.34 | 106.587 |
| -Domesticated species | 4516.31 | 99.557 |

**Table S2. The alternate best fit multivariable zero-inflated negative binomial model predicting the number of zoonotic viruses in mammalian species**. The alternate zero-inflated negative binomial regression model evaluating variation in zoonotic virus richness among extant terrestrial mammalian species is shown with model parameters indicating relative importance (IRR) and significance (with 95% confidence interval) for all variables. All variables significantly associated with the number of zoonotic viruses in a host species in the zero-inflated negative binomial model were significantly related to zoonotic virus richness except for one category of criteria for listing of species as threatened.

| **Variables** | **IRR**† | **95% Confidence Interval** | **P-Value** |
| --- | --- | --- | --- |
| **Data Deficient** | **0.268** | **(0.21, 0.33)** | **< 0.001** |
| **Conservation Status‡** |  |  |  |
| Least concern increasing | 1.645 | (1.04, 2.6) | 0.033 |
| Least concern decreasing | 0.566 | (0.41, 0.78) | 0.001 |
| Near threatened decreasing | 0.267 | (0.17, 0.43) | < 0.001 |
| Vulnerable threatened status | 0.132 | (0.07, 0.25) | < 0.001 |
| Endangered threatened status | 0.104 | (0.05, 0.21) | < 0.001 |
| Critically endangered threatened status | 0.058 | (0.02, 0.15) | < 0.001 |
| **IUCN Criteria for Threatened Status§** |  |  |  |
| Population size reduction by direct observation (A1, A2, A4(a)) | 3.667 | (1.8, 7.49) | < 0.001 |
| Decline in area of occupancy or habitat quality (A1-4(c)) | 1.816 | (0.88, 3.73) | 0.104 |
| Population size reduction based on levels of exploitation (A1-4(d)) | 2.01 | (1.04, 3.88) | 0.037 |
| Small extent of occurrence (B1) | 0.236 | (0.08, 0.69) | 0.008 |
| **Taxonomic Order\|\|** |  |  |  |
| Primates | 2.348 | (1.74, 3.16) | < 0.001 |
| Chiroptera | 1.698 | (1.36, 2.12) | < 0.001 |
| Diprotodontia | 0.234 | (0.1, 0.56) | 0.001 |
| Eulipotyphla | 0.166 | (0.08, 0.32) | < 0.001 |
| **Domesticated Species** | **11.821** | **(3.69, 37.84)** | **< 0.001** |

* The zero-inflation negative binomial model incorporates the ln PubMed hits as a continuous variable predicting excess zeros (OR 0.31, 95% CI 0.22-0.42, p < 0.001). This zero-inflated negative binomial model showed less overall fit (McFadden’s R^2^ = 0.184) compared to the zero-inflated Poisson model.

†The incident rate ratio (IRR) reflects the relative influence on the expected number of zoonotic viruses in a given species for a given category compared to the reference category specified. This model incorporates a logit model to predict non-detections in host species designated with ‘data deficient/unknown population trend’.

‡Compared to least concern, stable

§Compared to all other criteria for listing as threatened, based on IUCN Red List Criteria used to evaluate whether species belong in a threatened category; for threatened species only (3)

||Compared to all other orders

**Table S3: The multivariable zero-inflated Poisson model predicting the number of zoonotic viruses in mammalian species without adjusting for number of publications in a species**. The best fit zero-inflated Poisson regression model evaluating variation in zoonotic virus richness among extant terrestrial mammalian species is shown with model parameters indicating relative importance (IRR) and significance (with 95% confidence interval) for variables in the final model except for ln PubMed hits.

| **Variables** | **IRR**† | **95% Confidence Interval** | **P-Value** |
| --- | --- | --- | --- |
| **Conservation Status‡** |  |  |  |
| Least concern increasing | 1.555 | (1.22, 1.98) | < 0.001 |
| Least concern decreasing | 0.677 | (0.53, 0.86) | 0.001 |
| Near threatened decreasing | 0.391 | (0.26, 0.58) | < 0.001 |
| Vulnerable threatened status | 0.128 | (0.07, 0.22) | < 0.001 |
| Endangered threatened status | 0.127 | (0.07, 0.23) | < 0.001 |
| Critically endangered threatened status | 0.057 | (0.03, 0.13) | < 0.001 |
| **IUCN Criteria for Threatened Status§** |  |  |  |
| Population size reduction by direct observation (A1, A2, A4(a)) | 3.331 | (2.07, 5.37) | < 0.001 |
| Decline in area of occupancy or habitat quality (A1-4(c)) | 2.127 | (1.22, 3.72) | 0.008 |
| Population size reduction based on levels of exploitation (A1-4(d)) | 2.340 | (1.39, 3.93) | 0.001 |
| Small extent of occurrence (B1) | 0.223 | (0.09, 0.58) | 0.002 |
| **Taxonomic Order\|\|** |  |  |  |
| Primates | 2.017 | (1.66, 2.44) | < 0.001 |
| Chiroptera | 1.284 | (1.1, 1.5) | 0.002 |
| Diprotodontia | 0.224 | (0.1, 0.51) | < 0.001 |
| Eulipotyphla | 0.142 | (0.07, 0.27) | < 0.001 |
| **Domesticated Species** | **8.734** | **(6.72, 11.34)** | **< 0.001** |

*Results shown are from the count model (Poisson with log link). The zero-inflation model (binomial with logit link) incorporates the data deficient/unknown population trend variable result as an odds ratio predicting excess zeros (OR 2.14, 95% CI 1.56-2.81, p < 0.001). This effect zero-inflated Poisson model showed good overall fit (McFadden’s R^2^ = 0.175).

†The incident rate ratio (IRR) reflects the relative influence on the expected number of zoonotic viruses in a given species for a given category compared to the reference category specified. This model incorporates a logit model to predict non-detections in host species designated with ‘data deficient/unknown population trend’.

‡Compared to least concern, stable

§Compared to all other criteria for listing as threatened, based on IUCN Red List Criteria used to evaluate whether species belong in a threatened category; for threatened species only (3)

||Compared to all other orders

**Data file S1.** List of zoonotic viruses and associated terrestrial mammalian hosts documented in peer-reviewed literature published through December 2013.

**Data file S2.** List of mammalian species, their taxonomic classification, their domestication status, and The International Union for Conservation of Nature (IUCN) Red List categories and criteria (3) used to evaluate their relationship with the number of unique zoonotic viruses recognized in a species as published through December 2013.

**References**

1. Taylor LH, Latham SM, Woolhouse ME. Risk factors for human disease emergence. Philos Trans R Soc Lond B Biol Sci. 2001;356(1411):983-9.

2. Jones KE, Patel NG, Levy MA, Storeygard A, Balk D, Gittleman JL, et al. Global trends in emerging infectious diseases. Nature. 2008;451(7181):990-3.

3. IUCN. The IUCN Red List of Threatened Species. Version 2017-1 ed2017.

4. Pandit PS, Doyle MM, Smart KM, Young CCW, Drape GW, Johnson CK. Predicting wildlife reservoirs and global vulnerability to zoonotic Flaviviruses. Nat Commun. 2018;9(1):5425.

5. Olival KJ, Hosseini PR, Zambrana-Torrelio C, Ross N, Bogich TL, Daszak P. Host and viral traits predict zoonotic spillover from mammals. Nature. 2017;546(7660):646-50.
